# Insights into the Spectrum of Activity and Mechanism of Action of MGB-BP-3

**DOI:** 10.1021/acsinfecdis.2c00445

**Published:** 2022-11-29

**Authors:** Charlotte Hind, Melanie Clifford, Charlotte Woolley, Jane Harmer, Leah M. C. McGee, Izaak Tyson-Hirst, Henry J. Tait, Daniel P. Brooke, Stephanie J. Dancer, Iain S. Hunter, Colin J. Suckling, Rebecca Beveridge, John A. Parkinson, J. Mark Sutton, Fraser J. Scott

**Affiliations:** † Research and Evaluation, UKHSA Porton Down, SalisburySP4 0JG, United Kingdom; ‡ School of Applied Sciences, University of Huddersfield, Queensgate, HuddersfieldHD1 3DH, United Kingdom; § Department of Pure and Applied Chemistry, University of Strathclyde, GlasgowG1 1XL, United Kingdom; ∥ Department of Microbiology, Hairmyres Hospital, NHS Lanarkshire, GlasgowG75 8RG, United Kingdom; ⊥ School of Applied Sciences, Edinburgh Napier University, EdinburghEH11 4BN, United Kingdom; # Strathclyde Institute of Pharmacy & Biomedical Sciences, University of Strathclyde, GlasgowG4 0RE, United Kingdom; ∇ Institute of Pharmaceutical Science, School of Cancer & Pharmaceutical Science, King’s College London, Franklin-Wilkins Building, 150 Stamford Street, LondonSE1 9NH, United Kingdom

**Keywords:** Strathclyde minor groove binders, DNA binding, synergy, Gram-positive, Gram-negative, topoisomerase

## Abstract

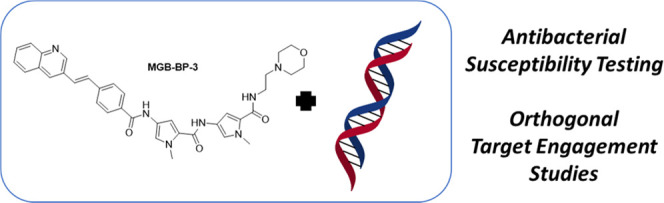

MGB-BP-3 is a potential first-in-class antibiotic, a Strathclyde Minor Groove Binder (S-MGB), that has successfully completed Phase IIa clinical trials for the treatment of *Clostridioides difficile* associated disease. Its precise mechanism of action and the origin of limited activity against Gram-negative pathogens are relatively unknown. Herein, treatment with MGB-BP-3 alone significantly inhibited the bacterial growth of the Gram-positive, but not Gram-negative, bacteria as expected. Synergy assays revealed that inefficient intracellular accumulation, through both permeation and efflux, is the likely reason for lack of Gram-negative activity. MGB-BP-3 has strong interactions with its intracellular target, DNA, in both Gram-negative and Gram-positive bacteria, revealed through ultraviolet–visible (UV–vis) thermal melting and fluorescence intercalator displacement assays. MGB-BP-3 was confirmed to bind to dsDNA as a dimer using nano-electrospray ionization mass spectrometry and nuclear magnetic resonance (NMR) spectroscopy. Type II bacterial topoisomerase inhibition assays revealed that MGB-BP-3 was able to interfere with the supercoiling action of gyrase and the relaxation and decatenation actions of topoisomerase IV of both *Staphylococcus aureus* and *Escherichia coli*. However, no evidence of stabilization of the cleavage complexes was observed, such as for fluoroquinolones, confirmed by a lack of induction of DSBs and the SOS response in *E. coli* reporter strains. These results highlight additional mechanisms of action of MGB-BP-3, including interference of the action of type II bacterial topoisomerases. While MGB-BP-3′s lack of Gram-negative activity was confirmed, and an understanding of this presented, the recognition that MGB-BP-3 can target DNA of Gram-negative organisms will enable further iterations of design to achieve a Gram-negative active S-MGB.

Drugs that target the minor groove of DNA, minor groove binders (MGBs), have been extensively investigated as anti-infective agents, including the targeting of bacterial, fungal, viral, and parasitic pathogens.^1^ One notable MGB is the natural product, distamycin, which has anti-infective and anticancer properties; however, its unfavorable cytotoxicity prevented its development as a drug.^2^ Distamycin binds to AT-rich sequences of dsDNA to inhibit the formation of transcription complexes.^3^

Strathclyde MGBs (S-MGBs), based upon a distamycin template, have shown remarkable anti-infective properties. Their favorable cytotoxicity profiles to mammalian cells give them selectivity indices that make them suitable for development as novel drugs. This has enabled extensive *in vitro* and several *in vivo* experiments to provide proof of concept for S-MGBs as a novel class of anti-infective agent against bacterial, fungal, viral, and parasitic infections.^4−12^ One of these compounds, MGB-BP-3 (Figure 1) has successfully completed Phase IIa clinical trials for the treatment of *Clostridioides difficile* associated disease (NCT03824795). MGB-BP-3 also has potent (<1 μg/mL) antibacterial activity against methicillin-resistant and methicillin-susceptible *Staphylococcus* spp., *Streptococcus* spp., and vancomycin-resistant and vancomycin-susceptible *Enterococcus* spp.^13^ There is limited information in the literature regarding MGB-BP-3′s activity against Gram-negative organisms, possibly reflecting the well-known challenges of penetration through the double membrane system.

**Figure 1:**
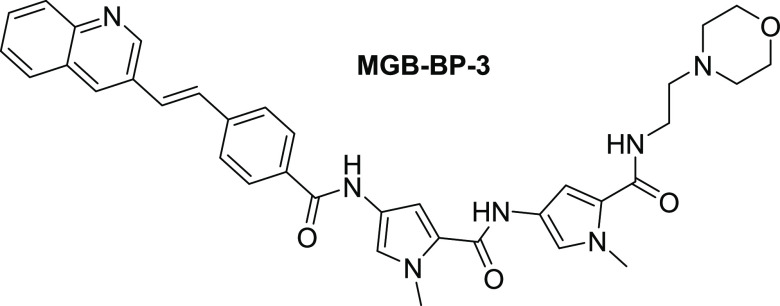
Structure of MGB-BP-3.

Dose-response curves of MGB-BP-3 against*Staphylococcus aureus* show a steep decrease in bacterial viability indicative of catastrophic failure of the biochemical machinery within the bacterium, rather than a sigmoidal dose-response curve indicative of interaction with a single molecular target.^13^ This is perhaps unsurprising as MGB-BP-3, and S-MGBs more generally, are thought to bind to many AT-rich sequences within the minor groove of dsDNA, similar to the template natural product distamycin. MGB-BP-3 is hypothesized to bind as a dimer, with two MGB-BP-3 molecules within each binding site, again, similar to distamycin. This binding mode has been shown for some early S-MGBs, and also more therapeutically interesting alkene-containing S-MGBs but not MGB-BP-3 itself.^14^ However, there have been limited studies that specifically characterize the interaction of MGB-BP-3 with dsDNA. The biological consequences of MGB-BP-3 interacting with dsDNA have recently been explored using RNA-Seq and DNase I and potassium permanganate footprinting. It was demonstrated that MGB-BP-3 binds to and inhibits transcription from multiple essential promoters on the *S. aureus* chromosome, and that resistant mutants were unable to be generated by serial passage experiments.^13^ However, given the molecule’s ability to bind to many different sites on the bacterial genome, MGB-BP-3 has the potential to interfere with other biological processes involving DNA, although these have not yet been investigated.

Herein, an investigation into the origin of MGB-BP-3′s selective activity against Gram-positive bacteria is presented, including synergy studies and fluorescence microscopy. Furthermore, we expand the evidence base for MGB-BP-3′s multiple mechanisms of action by providing a detailed account of its interaction with dsDNA and interference of the action of type II bacterial topoisomerases. Studies also explore reasons for the lack of significant Gram-negative activity to determine whether there are prospects of developing S-MGBs for these species.

## Results and Discussion

### MGB-BP-3 Has Significant Activity against Gram-Positive Bacteria but not against Gram-Negatives Due to Poorer Accumulation

The selective activity of MGB-BP-3 for only Gram-positive bacteria was confirmed using a panel of ESKAPE pathogens comprising two Gram-positive (*S. aureus* and *Enterococcus faecalis*) and four Gram-negative (*E. coli*, *Pseudomonas aeruginosa*, *Acinetobacter baumannii*, and *Klebsiella pneumoniae*) isolates (Table 1). As expected, MGB-BP-3 had potent activities against the Gram-positive bacteria (MIC_80_ 0.2 μM) but no measurable MICs (up to 100 μM) against the Gram-negative bacteria. Potent activity against Gram-positive pathogens was further demonstrated using an expanded panel of Gram-positive strains (*N* = 7, MIC range: 0.1–0.78 μM, Table S1).

**Table 1:** Activity of MGB-BP-3 against ESKAPE Pathogens and Potentiation Results with PAβN against Gram-Negative Pathogens Using Checkerboard Assays^a^

	*S. aureus* ATCC 43300	*E. faecalis* ATCC 51299	*E. coli* ATCC 25922	*P. aeruginosa* ATCC 27893	*A. baumannii* ATCC 19606	*K. pneumoniae* ATCC 700603
MIC_80_ (μM) MGB-BP-3	0.2	0.2	>100	>100	>100	>100
MIC_80_ (μM) MGB-BP-3 with 100 μg/mL PAβN	NT	NT	0.05	0.2	0.1	0.78
FICI MGB-BP-3 and PAβN	NT	NT	≤0.03	≤0.1	≤0.1	≤0.2

a Fractional inhibitory concentration indices (FICIs) from checkerboard assays indicate significant synergy for values <0.5.

**Figure 2:**
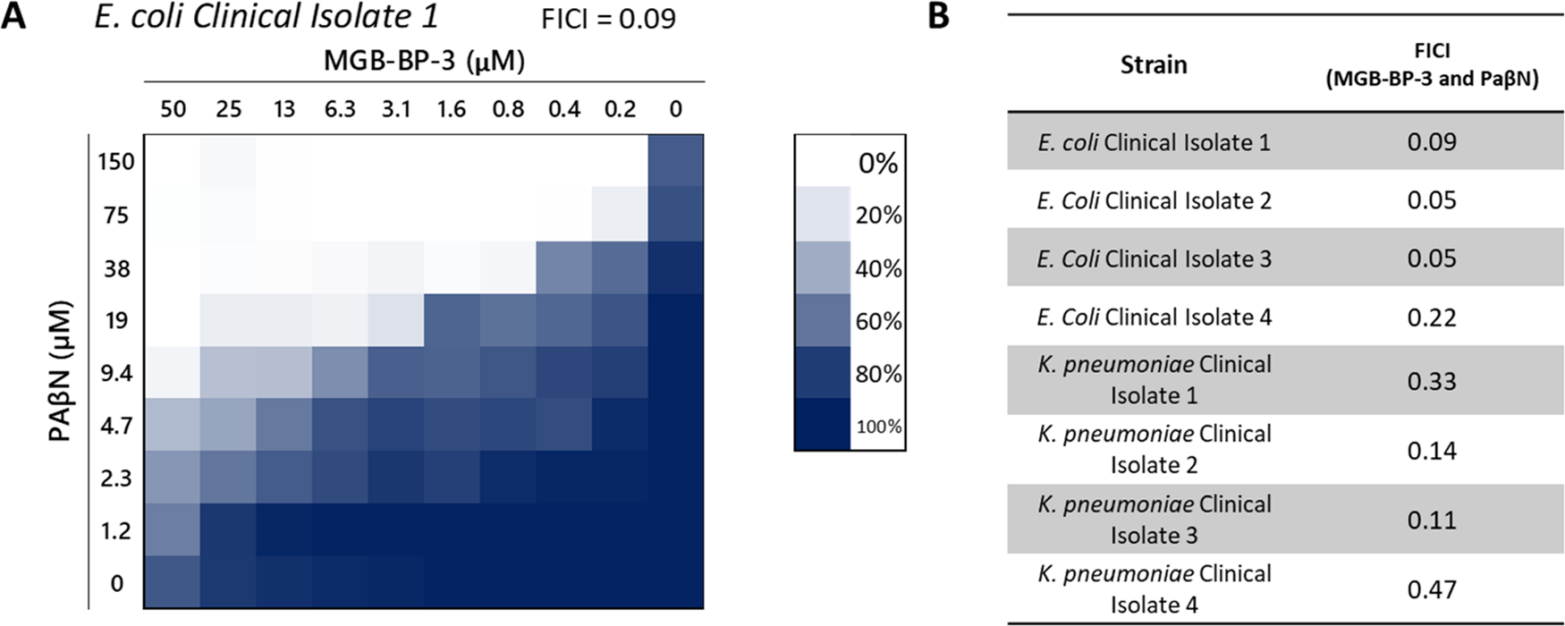
Checkerboard assays of MGB-BP-3 and PAβN against clinical isolates of Gram-negative pathogens. Fractional inhibitory concentration indices (FICIs) from checkerboard assays indicate significant synergy for values <0.5. (A) Exemplar visualization of the checkerboard assay against a clinical isolate of *E. coli*. (B) Calculated FICIs for all clinical isolates tested against.

To provide initial insight into the lack of Gram-negative activity, synergy between MGB-BP-3 and the efflux pump inhibitor and membrane permeabilizer, phenylarginine β-naphthylamide (PAβN), was investigated (Table 1). A high concentration of PAβN (100 μg/mL), with assay conditions lacking Mg ions, was used in this initial experiment to afford both efflux inhibition and membrane permeabilization.^15^ In combination with PAβN, MGB-BP-3 had potent MICs, less than 1 μM, against all Gram-negative bacteria tested. Reduction in MICs ranged from at least 100-fold (*K. pneumoniae*) to more than 2000-fold (*E. coli*) compared with MGB-BP-3 alone. This synergism was confirmed by carrying out checkerboard assays using the same set of Gram-negative bacteria. Fractional inhibitory concentration indices (FICIs) were indicative of strong synergy between MGB-BP-3 and all Gram-negative bacteria (Table 1). Checkerboard assays using a small panel of multidrug-resistant clinical isolates of *E. coli* and *K. pneumoniae* confirmed synergism (Figure 2).

**Figure 3:**
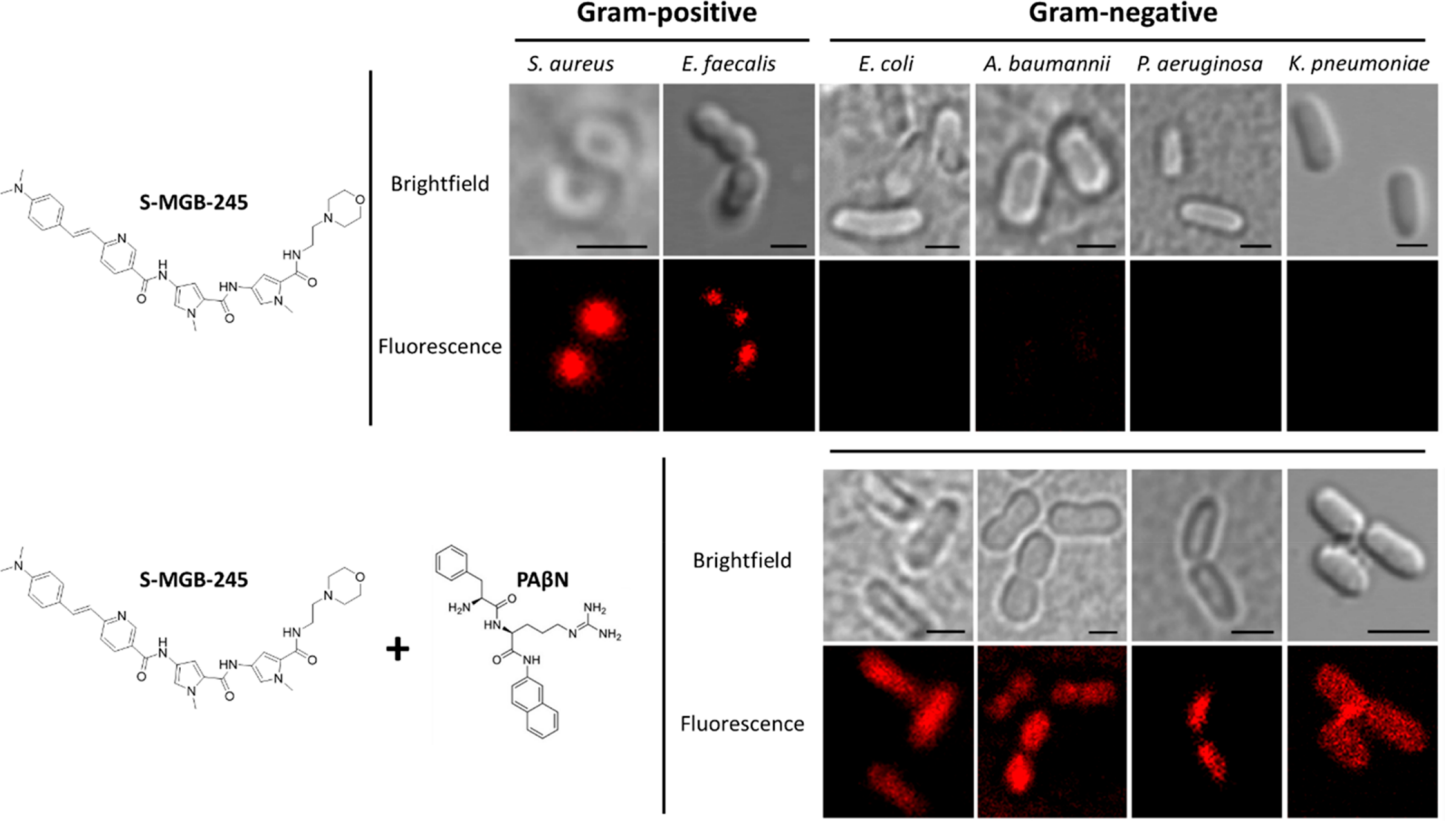
ESKAPE pathogens were treated with fluorescent S-MGB-245 (1 μM) and viewed using confocal microscopy. The S-MGB only accumulates the Gram-positive organisms (top panels). Co-treatment with PAβN (100 μg/mL) enables intracellular accumulation of S-MGB in Gram-negative cells (bottom panels).

These results are indicative of the intrinsic resistance of Gram-negative bacteria to MGB-BP-3 being due to poor intracellular accumulation. To further demonstrate this, a fluorescent analogue of MGB-BP-3, S-MGB-245, which has a similar lack of Gram-negative activity (Table S2), was used in fluorescence microscopy studies of the same panel of ESKAPE pathogens (Figure 3). When used alone, S-MGB-245 can be seen to accumulate only in the cells of the Gram-positive bacteria. However, when used in combination with PAβN at permeabilizing and efflux inhibiting concentrations (50 μM, no Mg ions), S-MGB-245 accumulates inside Gram-negative bacteria. These fluorescence imaging studies, along with the previous synergy data, provide strong evidence that MGB-BP-3′s lack of activity against Gram-negative organisms is due to insufficient intracellular accumulation. The energy dependence of S-MGB-245′s uptake in *S. aureus* was also investigated using sodium azide as an energy poison. S-MGB-245 was still observed inside cells, suggesting that uptake in *S. aureus* is not *via* an active transport-mediated route (Figure S1).

### Poor Intracellular Accumulation of MGB-BP-3 in Gram-Negative Organisms May Be due to Both Poor Uptake and Efficient Efflux

Poor intracellular accumulation of MGB-BP-3 is likely to be caused by either efficient efflux, poor membrane permeation, or a combination thereof. To investigate these possibilities, we carried out potentiation studies on a range of well-characterized Gram-negative bacteria using the membrane permeabilizer, polymyxin B nonapeptide (PMBN), and the efflux pump inhibitor PAβN used in the presence of Mg to minimize its membrane permeation effects.^15^ As expected, no significant activity was observed in any of the strains tested without PMBN or PAβN (Table 2). For *A. baumannii* and *E. coli*, both PMBN and PAβN significantly reduced the MIC, suggesting that the low basal activity is a function of both poor uptake across the membranes and the potential for active efflux through one or more efflux pump systems. There were differences observed in the effects for two other strains, *P. aeruginosa* NCTC 13437 and *K. pneumoniae* NCTC 13368, showing no potentiation with PMBN or PAβN. This suggests that neither blockage of efflux through Resistance Nodulation Division (RND) family pumps (reflecting published PAβN specificity) nor membrane permeation is sufficient in isolation to potentiate activity in these strains. There could also be other factors that contribute to elevated resistance.^16−18^ Activity was potentiated in strains known to be more permeable, *K. pneumoniae* M6 (PMBN and PAβN) and PAO1 (PMBN only). The latter again suggests that inhibition of RND efflux pumps in *P. aeruginosa* is not enough to potentiate activity.

**Table 2:** MICs of MGB-BP-3 against Gram-Negative ESKAPE Pathogens in the Presence and Absence of PMBN (30 μg/mL) and PAβN (25 μg/mL in 0.04 mM MgSO4)^a^

			MGB-BP-3 (μM)		
organism	strain		+PMBN	+PaβN	CIP (μg/mL)
*K. pneumoniae*	NCTC 13368	>100	>100	>100	0.5
*K. pneumoniae*	M6	>100	3.13	1.56	≤0.125
*A. baumannii*	AYE	>100	3.13	6.25	64
*A. baumannii*	ATCC 17978	>100	0.78–3.13	0.78–3.13	0.5
*P. aeruginosa*	PA01	>100	0.39–3.13	>100	0.5
*P. aeruginosa*	NCTC 13437	>100	>100	>100	64
*E. coli*	NCTC 12923	>100	0.78	0.78	≤0.125

a Ciprofloxacin (CIP) was included as a control antibiotic.

The role of efflux in mediating resistance was confirmed using directed insertion mutants from the Keio collection.^19^ Although the effects are likely to be pleiotropic, a *tolC* mutant showed a significant reduction in the MIC suggesting that efflux through AcrAB-TolC, or other membrane transporters that utilize TolC, is a component of reduced efficacy in Gram-negative bacteria (Table 3). Similar effects were observed for ciprofloxacin, another AcrAB-TolC substrate. This corresponds with the observed potentiation with PAβN in both *E. coli* and *K. pnuemoniae* strains (Table 2). Conversely, the tested insertion mutant in *surA*, the chaperone associated with the β-barrel assembly machine (Bam) complex, resulted in significantly higher MICs for MGB-BP-3 but not for ciprofloxacin, suggesting that the observed reduction in susceptibility is perhaps linked to specific features of the β-barrel protein complement of the outer membrane (Table 3). The MIC plates showed a very specific response for the *surA* knockout mutant, with a significant reduction in growth at 1.56–3.13 μM, but a long trailing endpoint with residual growth observed up to a concentration of 100 μM. This could relate to changes in membrane localization of specific permeases or porins, affecting uptake into the cell and/or may reflect two different mechanisms of actions within the *surA* mutant strain. The MIC for rifampicin, which cannot easily pass through the Gram-negative outer membrane, was essentially unchanged in this strain (within 2-fold of the MIC for the parental strain), suggesting that the membrane itself remains intact in this directed insertion mutant under the conditions tested.

**Table 3:** MICs of MGB-BP-3 and Relevant Control Antibiotics against the *surA* and *tolC* Mutants from the *E. coli* Keio Collection

		MGB-BP-3 (μM)		
		MIC	CIP MIC (μg/mL)	RIF MIC (μg/mL)
JW0052	*surA* knockout (membrane)	>100	0.02	8
JW5503	*tolC* knockout (efflux)	0.39	0.004	2
BW25113	WT	6.25	0.02	4

To provide more direct evidence for why MGB-BP-3 demonstrates poor uptake, we used an LPS competition assay with *S. aureus* as a bioassay, given its high degree of susceptibility. The data (Table 4) confirmed that LPS derived from *E. coli* was able to significantly increase the MIC of MGB-BP-3, but this was not the case for the control antibiotic vancomycin, which does not have a strong affinity for LPS. This suggests that MGB-BP-3′s poor uptake by Gram-negative bacteria may, in part, be due to sequestration by LPS.

**Table 4:** MIC of MGB-BP-3 and Vancomycin against *S. aureus* ATCC 43300 in the Presence of 1 μg/mL *E. coli* LPS

	*S. aureus* ATCC 43300 MIC_80_ (μM)	
condition	MGB-BP-3	vancomycin
drug only	0.1	0.2
drug and LPS	0.8	0.2
fold-increase	8	1

Collectively, these experiments suggest that poor intracellular accumulation of MGB-BP-3 in Gram-negative organisms may be due to both poor uptake and efficient efflux. The precise mechanism has yet to be identified; however, the data presented here suggest that it may be strain-specific.

### MGB-BP-3 Binds to DNA

The binding of MGB-BP-3 to dsDNA was explicitly demonstrated using a number of different methods; these included thermal melt analysis of genomic DNA (gDNA) of salmon, a fluorescence intercalator displacement assay using gDNA of the ESKAPE pathogens, a native mass spectrometry experiment using a short AT-rich DNA oligomer, and an NMR investigation of MGB-BP-3 binding to the same short oligomer.

#### Thermal Melt of Salmon gDNA

**Figure 4:**
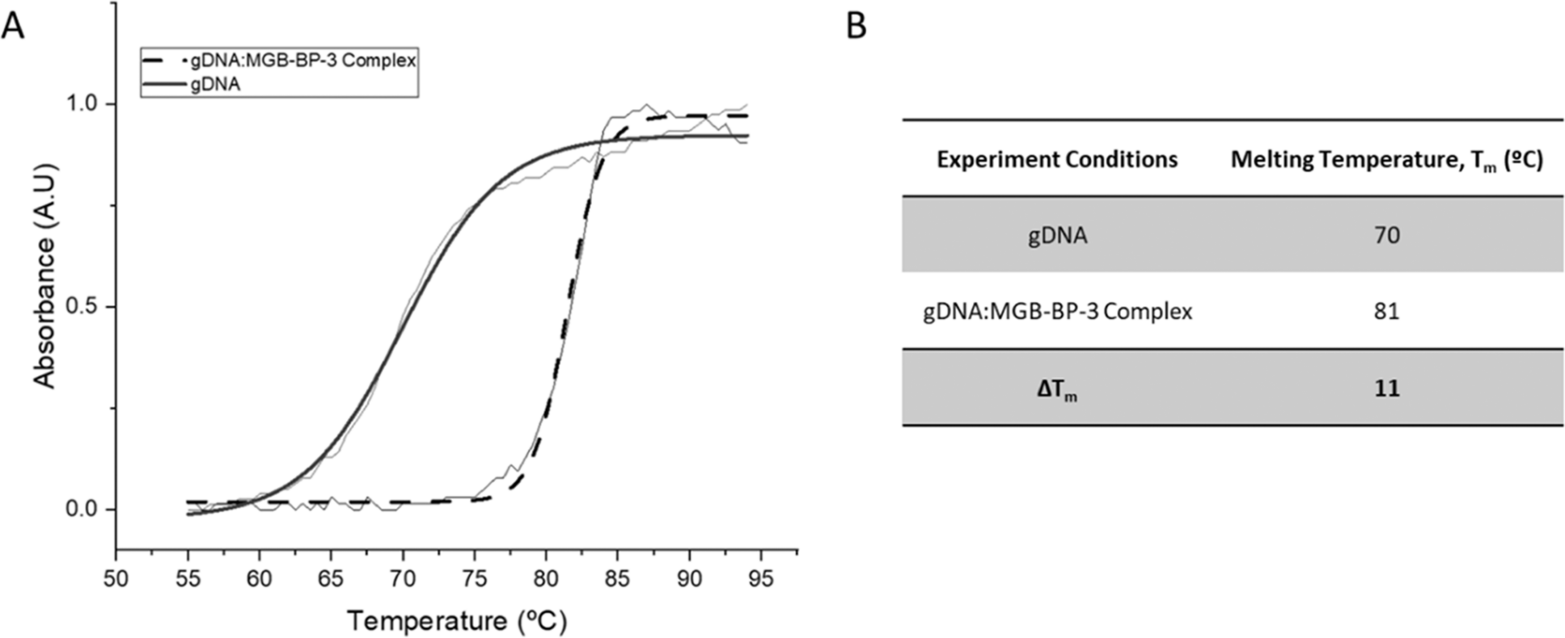
Thermal melt curves of gDNA (salmon) and gDNA:MGB-BP-3 Complex. (A) Exemplar melt curve from one experimental repeat, visually representing the different melt curves of gDNA and the gDNA:MGB-BP-3 Complex. Data has been fitted with a Boltzmann distribution. (B) Melting temperatures of gDNA and gDNA:MGB-BP-3 Complex calculated from fitted Boltzmann distributions using OriginPro 2021. All values are an average for *n* = 4 experimental repeats with an error of ±1 °C.

Thermal melt analysis of MGB-BP-3 binding to salmon gDNA showed significant stabilization of the complex, indicative of strong binding (Δ*T*_m_ = 11 °C, Figure 4).

#### Fluorescence Intercalator Displacement Assay on Bacterial gDNA

The binding of MGB-BP-3 to gDNA extracted from the two Gram-positive and four Gram-negative bacteria used for the previously described screening, synergy, and fluorescence microscopy studies (Table 1 and Figure 3) was investigated using a fluorescence intercalator displacement (FID) assay. MGB-BP-3 bound to the gDNA of all six bacteria to a similar extent as each other, and to salmon gDNA (Table 5). This indicates that both Gram-positive and Gram-negative bacterial gDNA are targets for MGB-BP-3, consistent with the argument that low intracellular accumulation gives rise to the lack of activity against Gram-negative bacteria.

**Table 5:** Remaining Fluorescence (%) of SybrSafe Probe upon Addition of MGB-BP-3 to gDNA^a^

	gDNA from different organisms						
	*S. aureus* ATCC 43300	*E. faecalis* ATCC 51299	*E. coli* ATCC 25922	*P. aeruginosa* ATCC 27893	*A. baumannii* ATCC 19606	*K. pneumoniae* ATCC 700603	salmon
fluorescence (%)	20 ± 4	28 ± 3	25 ± 2	27 ± 3	19 ± 1	23 ± 4	22 ± 2

a 0% indicates complete displacement of the probe, strong binding of MGB-BP-3 and 100% indicates no displacement of the probe, and no binding of MGB-BP-3 (*N* = 3).

#### nESI-MS on 5′-d(CGCATATATGCG)-3′

**Figure 5:**
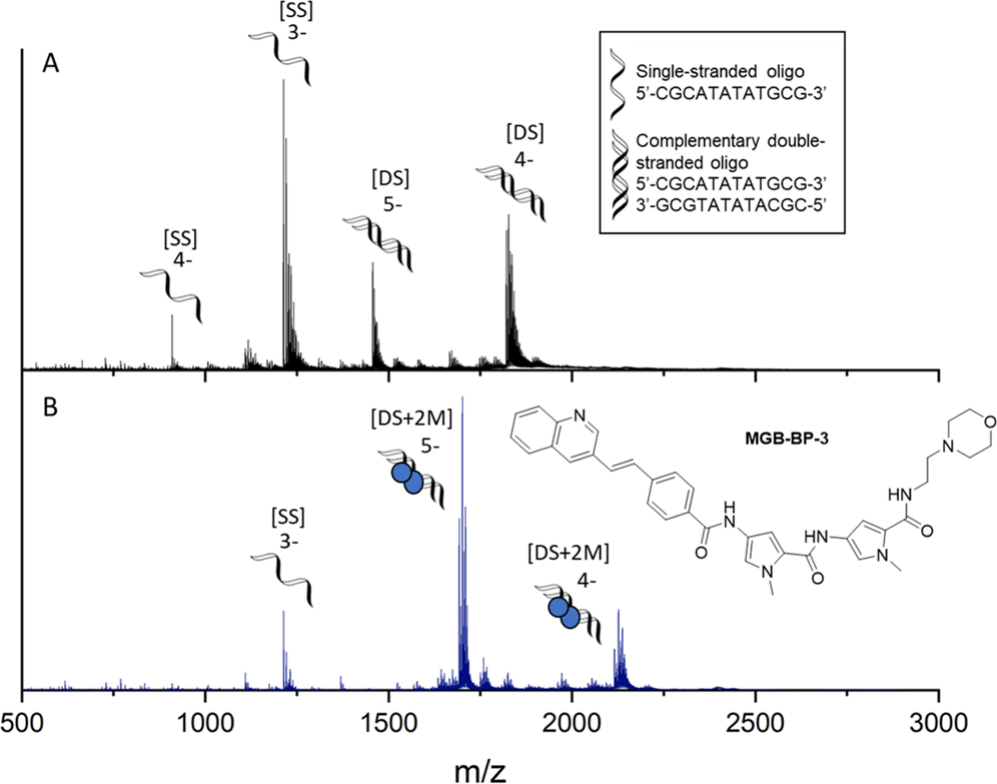
Characterization of MGB-BP-3 binding to double-stranded DNA as a dimer. nESI-MS of DNA sequence 5′-CGCATATATGCG-3′ (9 μM DNA, 100 μM KCl, 1% DMSO) sprayed from ammonium acetate (150 mM, pH 7) in the absence (A) and presence (B) of 100 μM MGB-BP-3. (A) Single-stranded DNA, [SS], is present in charge states 4- and 3-, and double-stranded DNA denoted [DS] is present in charge states 5- and 4-. (B) [DS] is only observed as a 2:1 complex with MGB-BP-3, with two molecules binding to each duplex (denoted [DS + 2M]), in charge states 5- and 4-.

Nano-electrospray ionization mass spectrometry (nESI-MS) was also used to confirm that MGB-BP-3 binds to dsDNA. Like the natural product distamycin, S-MGBs are thought to bind to AT-rich sequences as a dimer, i.e., two molecules of S-MGB bind within each binding site on the minor groove of dsDNA. For these experiments, a self-complementary oligomer with an AT-rich binding site (5′-CGCATATATGCG-3′) was used. nESI-MS of the oligomer alone shows the expected DNA duplex in charge states 4- and 5- (Panel A, Figure 5), and upon addition of MGB-BP-3, a complex is observed corresponding to the mass-to-charge ratio of two molecules binding to each duplex [DS + 2M], also in charge states 5- and 4- (Figure 5 and Table S4).

#### NMR Spectroscopy on 5′-d(CGCATATATGCG)-3′

**Figure 6:**
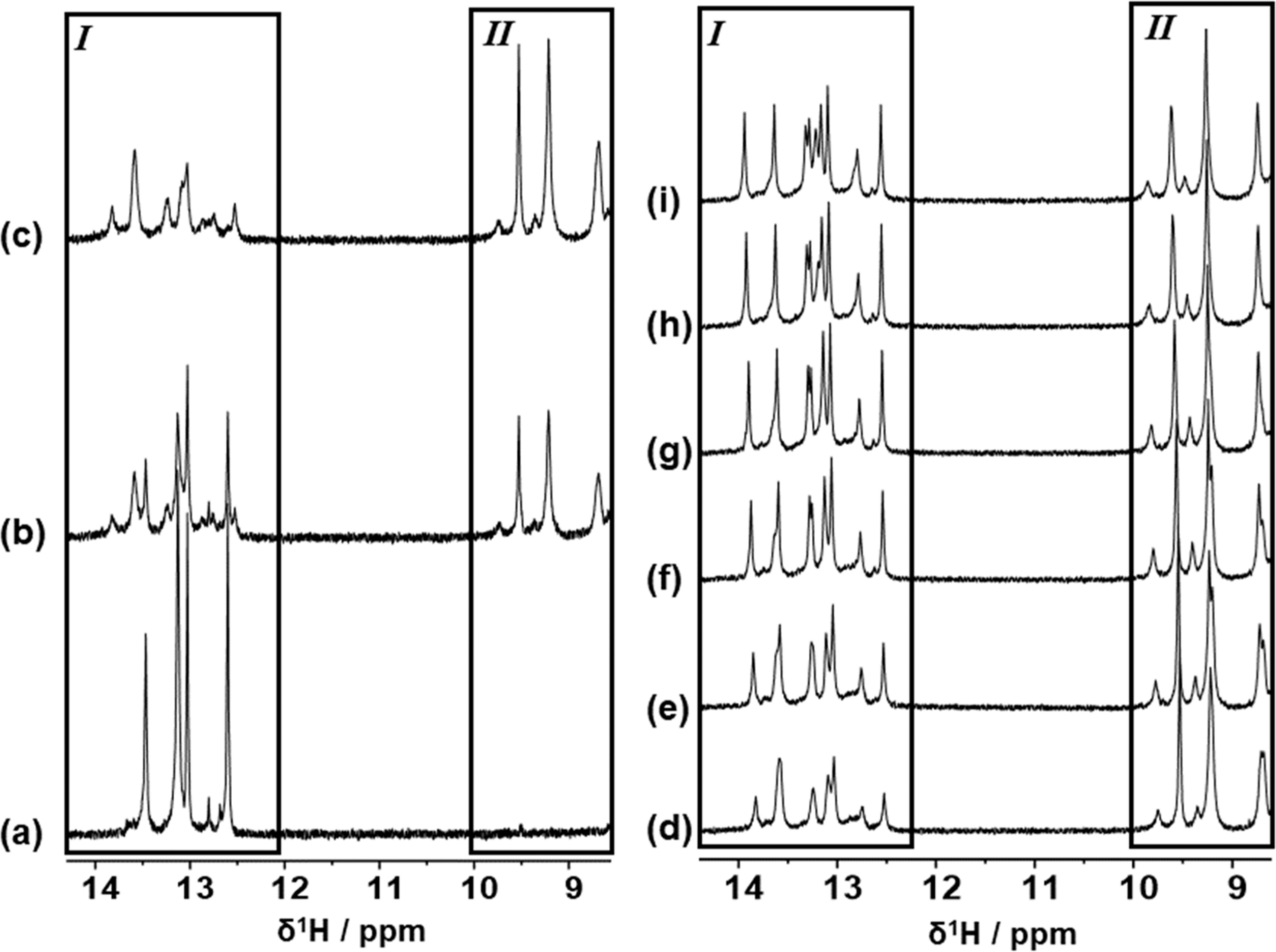
^1^H NMR spectroscopic evidence of association between DNA sequence 5′-CGCATATATGCG-3′ and MGB-BP-3. (a–c) High chemical shift regions of 600 MHz 1D ^1^H NMR spectra showing the results of MGB-BP-3 titration against DNA at 298 K under different molar ratio conditions of MGB-BP-3 to DNA duplex: (a) 0:1; (b) 1:1; and (c) 2:1. (d–i) High chemical shift regions of 800 MHz 1D ^1^H NMR spectra showing the results of gradual cooling of the 2:1 MGB-BP-3/DNA complex solution: (d) 298 K; (e) 293 K; (f) 288 K; (g) 283 K; (h) 278 K; and (i) 274 K. Regions shown boxed and labeled as I show proton NMR resonances arising from DNA imino protons; regions shown boxed and labeled as II show proton NMR resonances arising from peptide NH protons within DNA-bound MGB-BP-3.

A one-dimensional ^1^H NMR spectrum was acquired on 5′-d(CGCATATATGCG)-3′ (50 mM phosphate buffer, 90% H_2_O/10% D_2_O, with TSP-*d*_4_). The free DNA showed the expected appearance of five imino proton signals in the region between δ^1^H = 12.50 and 13.50 ppm, integrating to 10 proton equivalents for 10 protons (five pairs of symmetrical protons) in slow chemical exchange from a potential total of 12 imino proton resonances (imino proton signals from “frayed” terminal base-pairs not observed) (Figure 6a and Table S5). The DNA sample was subsequently titrated with aliquots of a concentrated solution of MGB-BP-3 initially to a 1:1 ligand/DNA duplex molar ratio (Figure 6b) and finally to the end point at a ligand/DNA duplex molar ratio of 2:1 (Figure 6c) indicated by complete loss of imino proton NMR signals arising from free DNA duplex being replaced by a set of lower-intensity imino proton NMR signals (Figure 6a–c, Box I and Table S6). Additionally, new ^1^H NMR signals were observed growing into the NMR spectrum in the region δ^1^H = 8.50–10.00 ppm with each ligand aliquot addition and growing to a maximum by the end point (Figure 6c, Box II) and indicative of the ligand peptide NHs.

Low-intensity, broad-lineshape resonances associated with the DNA imino protons of a ligand/DNA complex are characteristic of looser association between the ligand and DNA, the formation of a less well-defined complex in terms of fit between the ligand and DNA compared with other complexes, suggestive of some conformational exchange, or a combination of some or all of these characteristics.^20^ To investigate this further, the 2:1 complex solution was cooled in steps of 5 °C from 25 to 5 °C and finally to 1 °C with ^1^H NMR spectra being recorded at each step (Figure 6d–i). Noteworthy was the sharpening and intensity increase of the DNA imino proton NMR resonances (Figure 6d–i, Box I). The improvement in the definition of these data upon sample cooling is characteristic of slower chemical exchange. At 800 MHz, it was possible to resolve eight unique imino proton NMR resonances with a further signal showing underlying evidence of two further imino proton NMR resonances, which was also supported by evidence from two-dimensional (2D) [^1^H, ^1^H] NOESY NMR data (Figure S2). At the lowest sample temperature, this region of the NMR data integrated to 10 proton equivalents, consistent with 10 different imino protons. Additionally, six methyl singlet NMR resonances were observed in the same NMR spectrum in a chemical shift window consistent with resonances arising from thymine methyl groups.

Without further supporting evidence, two possible explanations may be speculated. These observations may first be explained by the presence of a completely asymmetric ligand–DNA complex. Since two equivalents of ligand are bound, this explanation would require association of the individual ligands to be bound to the DNA in quite different locations or with quite different orientations or conformations with respect to each other. Alternatively, the observations may be explained by the presence of two different symmetrical ligand/DNA complexes, each of which gives rise to five imino proton resonances representing 10 imino protons for each symmetrical complex.

**Figure 7:**
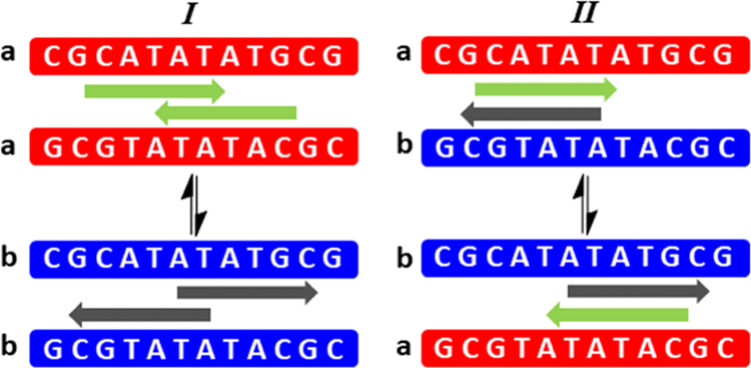
Potential binding models for the complex between MGB-BP-3 and DNA. Model I—two symmetrical DNA complexes coexist within the same sample and exchange their identities through ligand rearrangement. Model II—An asymmetric DNA complex in which the original symmetry of the free DNA duplex is lifted through ligand binding in a manner that confers different identities on each DNA strand within the duplex. In both cases, slow chemical exchange on the NMR chemical shift timescale is identified through an extensive set of chemical exchange cross-peaks within the NMR data.

To explore these possible explanations further, 800 MHz 2D [^1^H, ^1^H] NOESY NMR data of the cooled (1 °C) 2:1 ligand/DNA duplex sample were partially assigned. The DNA proton ^1^H NMR resonances for the 2:1 ligand/DNA complex indicate two uniquely identifiable sets of linked spatial correlations consistent with a typical data assignment protocol (see Figure S2). Chemical exchange links the two sets of data even at the temperature used to acquire these NMR data. The data may be interpreted by considering two possible ligand binding models (Figure 7). The first (Figure 7, I) suggests two different but symmetrical complexes existing simultaneously but identifiably within the same sample and interchanging with one another. The second (Figure 7, II) suggests an asymmetric complex existing, whereby the DNA strands themselves have different identities within the complex but which interchange owing to rearrangement of the ligands. Distinguishing between these two states requires the nature of the interaction between bound ligands and between bound ligand and DNA to be understood in detail, the work that is currently in progress and will be reported in a separate article. Despite the ambiguity between potential binding states, it is clear that the ligand forms a strong binding complex with DNA that undergoes slow exchange on the NMR chemical shift timescale.

### MGB-BP-3 Interferes with the Action of DNA Processing Enzymes *In Vitro*, but is Distinct from the Effect Seen with Fluoroquinolones

Having definitively demonstrated that MGB-BP-3 interacts strongly with dsDNA, the ability of this interaction to interfere with critical DNA processes was investigated. While it has been demonstrated that MGB-BP-3 can interfere with transcription,^13^ other DNA processes have not been investigated but remain possible contributors to the mechanisms of action of MGB-BP-3. The effects of MGB-BP-3 on type II bacterial topoisomerases, gyrase, and topoisomerase IV, in both *S. aureus* and *E. coli*, were investigated. There are many clinically relevant topoisomerase inhibitors, such as the fluoroquinolones, that poison the enzyme by shifting the DNA cleavage-religation equilibrium toward DNA cleavage, an increase in double-strand breaks (DSBs) and cell death.^21,22^ Thus, it was important to investigate if this mechanism was relevant to MGB-BP-3.

MGB-BP-3 interfered with the supercoiling action of gyrase and the relaxation and decatenation by topoisomerase IV (Table 6). It is notable that the effects of MGB-BP-3 are approximately the same toward the action of *S. aureus* and *E. coli* enzymes, further suggesting that the principal reason for the lack of Gram-negative activity is due to inefficient intracellular accumulation. The lack of activity in the gyrase and topoisomerase IV cleavage assays suggests that MGB-BP-3 does not cause the accumulation of DSBs as is observed in the mechanism of action of fluoroquinolones. This is further confirmed by the limited effects (<1.5-fold) of MGB-BP-3 on fluoroquinolone-resistant gyrases (*E. coli* S83L gyrase and *S. aureus* S84L gyrase mutants) on the supercoiling assay IC_50_s.

**Table 6:** Interference with the Action of DNA Processing Enzymes by MGB-BP-3

	*S. aureus*		*E. coli*	
enzyme	MGB-BP-3	fluoroquinolone^a^	MGB-BP-3	fluoroquinolone^a^
gyrase supercoiling IC_50_ (μM)	1.94	14.81	6.00	0.34
fluoroquinolone-resistant^b^ gyrase supercoiling IC_50_ (μM)	3.04	338.27	8.17	8.84
gyrase cleavage CC_50_ (μM)	>500	3.22	>500	0.29
Topo IV decatenation IC_50_ (μM)	2.50	7.69	1.93	1.82
Topo IV relaxation IC_50_ (μM)	2.02	0.86	1.98	2.44
Topo IV cleavage CC_50_ (μM)	>500	6.2	>500	6.2

a Ciprofloxacin control for all assays except the cleavage assays for which norfloxacin was used.

b S83L gyrase for *S. aureus* and S84L gyrase for *E. coli*.

**Figure 8:**
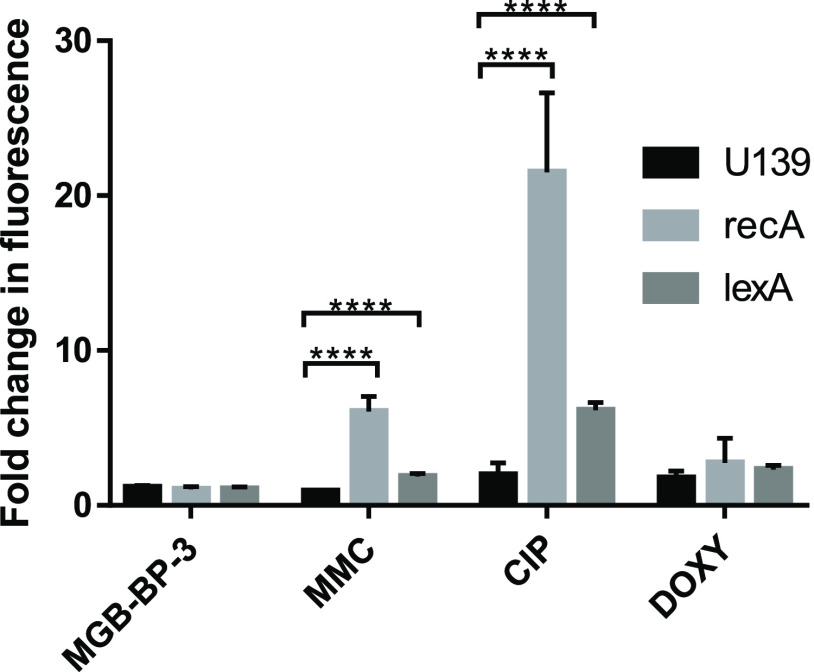
Fluorescence reporter strains from the K12 MG1655 library demonstrate that MGB-BP-3 has a different mechanism of action than the fluoroquinolones, as it does not activate the DNA SOS response in the same manner. These data are representative of three independent repeats. Error bars represent the standard deviation from the mean. ****refers to a *P* value of <0.0001, as assessed using unpaired *t*-tests.

To provide further evidence that the interference of the action of type II topoisomerases occurred through a mechanism distinct from fluoroquinolones, the SOS response was assessed in a series of *E. coli* reporter strains, as used previously (Figure 8).^18^ MGB-BP-3 has an MIC of 3.125 μM against the WT *E. coli* of the K12 MG1655 promoter library when tested in minimal media for this assay; however, in rich media, the MIC was >100 μM, matching the inactivity seen in other Gram-negative strains. A known inhibitor of type II topoisomerases (ciprofloxacin) and a DNA-damaging agent (mitomycin C (MMC)) were used as positive controls for the DNA SOS response, and a compound that interacts with the 30S subunit of the ribosome (doxycycline) was used as a negative control, all treated at 0.5× MIC. A significant fold-induction of fluorescence for *recA* and *lexA* was observed for ciprofloxacin and MMC, while no significant induction was seen for doxycycline. MGB-BP-3 did not induce the SOS response in this assay at 0.5× MIC, with no induction seen for either gene, comparable to doxycycline, which is known to kill bacteria by a mechanism not linked to DNA damage.^23^ This experiment confirms that, unlike the fluoroquinolones, while interference with the action of type II topoisomerase is likely to be a contributory part of the mechanism of action of MGB-BP-3, it does not induce DSBs and the SOS response.

Further evidence comes from the observation that MGB-BP-3 is equally effective with ciprofloxacin-resistant and ciprofloxacin-susceptible strains (Table S1), confirming the different mechanism of action.

## Conclusions

MGB-BP-3 is a member of a new class of drugs with a new mechanism of action and satisfies the four WHO criteria for genuine novelty.^24^ It is therefore important not only for the development of MGB-BP-3 itself as it addresses a Phase III clinical trial (NCT03824795) but also for the new class of drugs as a whole, that the best possible understanding of the mechanism of action is obtained.

The literature data supporting the development of MGB-BP-3 provide strong evidence for its effectiveness against Gram-positive pathogens; however, there is limited information on its effectiveness against Gram-negative pathogens. In this study, we add to the evidence that MGB-BP-3 is highly active against Gram-positive bacteria while demonstrating its limited activity against Gram-negative bacteria. As is the case for many Gram-positive only drugs, MGB-BP-3 appears to be ineffective against Gram-negative bacteria due to poor intercellular accumulation. This may be due to a combination of poor penetration and efficient efflux, but the specific mechanism appears to vary depending on the characteristics of the specific strain. In many cases, strong potentiation with PAβN and/or PMBN was observed that suggests that MGB-BP-3 may be effective against Gram-negative bacteria if an appropriate synergistic partner can be found, or its structure can be altered to allow for sufficient accumulation.

The full mechanism of action of MGB-BP-3 is not well understood, not least because its design adopts a multitargeted approach. Multiple experimental techniques have confirmed that MGB-BP-3 binds strongly to dsDNA. Importantly, by confirming that MGB-BP-3 binds to gDNA from both Gram-positive and Gram-negative organisms, we have provided further evidence that the difference in activity of MGB-BP-3 in these bacteria is due to differential intracellular accumulation, and not a difference in target engagement. The precise interactions of MGB-BP-3 with DNA were further characterized by demonstrating that the compound binds as a 2:1 dimer, similar to the natural product distamycin, from which it is derived. Furthermore, NMR studies strongly suggest the potential of a dynamic binding interaction with dsDNA, at least for the specific short, AT-rich dsDNA oligomer used in the experiments.

Previous studies employing RNA-Seq identified ∼700 transcripts with drug-altered expression profiles, many of which were associated with DNA replication, supercoiling, and primosome formation. Moreover, potassium permanganate footprinting confirmed that MGB-BP-3 binds to certain SigA-dependent promoter regions, preventing transcription of these genes.^13^ Here, MGB-BP-3 has been shown to interfere with other DNA-centric processes, such as the action of topoisomerase and gyrase, but it is not thought that MGB-BP-3 interacts with these enzymes directly to achieve this interference. Instead, we suggest that this is likely to be achieved by either MGB-BP-3 directly masking the enzyme binding site on DNA or indirectly altering the topology of the binding site through an allosteric mechanism from proximal binding. Notably, the absence of interference with the cleavage assays and the absence of induction of *recA* and *lexA* indicate a different mechanism of action to the fluoroquinolones, which is consistent with MGB-BP-3 binding to DNA and interfering with the formation of the DNA–enzyme complex.

Our study has contributed to the understanding of the mechanism of action of MGB-BP-3 and has identified additional DNA-centric mechanisms that MGB-BP-3 can interfere within bacteria. These findings provide further evidence that the resilience to resistance of MGB-BP-3 seen in the laboratory may be due to its multitargeted design requiring many simultaneous mutations at the multiple target sites on the genomic DNA.^13^ Consequently, the evolution of target-based resistance for MGB-BP-3, and by extension other S-MGBs, should be unlikely.

## Methods

### S-MGB Compounds

MGB-BP-3 and S-MGB-245 were prepared as previously described in refs (4) and (11), respectively.

### UV–Vis DNA Thermal Melting Experiments

Salmon DNA (D1626, Sigma-Aldrich) at 1 mg/mL in 1 mM phosphate buffer (pH 7.4) containing 0.27 mM KCl and 13.7 mM NaCl (P4417, Sigma-Aldrich) was annealed at 90 °C for 10 min. MGB-BP-3 at 10 mM in DMSO was diluted with the same phosphate buffer, and combined with the salmon DNA stock to yield a single sample with 10 μM S-MGB and 0.02 mg/mL gDNA in 1 mM phosphate buffer containing 0.27 mM KCl and 13.7 mM NaCl. Control samples containing only MGB-BP-3 or gDNA were prepared, respectively. Samples were melted at a rate of 0.5 °C/min from 45 to 90 °C with spectra recorded at 260 nm on a UV-1900 UV–vis spectrophotometer fitted with a Peltier temperature controller (Shimadzhu) using LabSolutions (Tm Analysis) software. The melting temperatures (Tms) of the MGB-BP-3:DNA complexes were determined by fitting a sigmoidal function using a Boltzmann distribution in OriginPro. Two independent experiments were carried out with values quoted with an error no worse than ±0.5 °C.

### Fluorescence Intercalator Displacement (FID) Method

Bacterial genomic DNA (ATCC 43300, DSM 13661; ATCC 27853, DSM1117; ATCC 700603, DSM 26371; ATCC 25922, DSM 1103; ATCC 19606, DSM 30007; ATCC 51299, DSM 12956; Leibniz Institute DSMZ-German Collection of Microorganisms and Cell Cultures GmbH) or salmon genomic DNA (deoxyribonucleic acid sodium salt from salmon testes, D1626, Merck) dissolved in 1 mM phosphate buffer pH 7.4 (containing 0.27 mM potassium chloride, 13.7 mM sodium chloride) to a concentration of 100 μg/mL in SybrSafe (SYBR Safe DNA Gel Stain, ×10,000 in DMSO, S33102 Invitrogen) was used as supplied by the manufacturer in DMSO, and MGB-BP-3 was prepared as 10 mM stock in DMSO. These stock solutions were diluted appropriately with each other and 1 mM phosphate buffer to give a test solution comprised of 20 μM S-MGB, 12,500-fold dilution of SybrSafe and 3.92 μg/mL DNA. Control solutions of gDNA and SybrSafe, gDNA, and SybrSafe at these concentrations were also prepared. Test and control solutions were heated to 30 °C and the fluorescence of each solution was measured using the SYBER filter setting of a StepOnePlus using melt analysis mode (StepOne Software v2.3). The reduction of fluorescence due to the binding of MGB-BP-3 to the gDNA was calculated as a normalized percentage based on the fluorescence measured due to the control with SybrSafe and gDNA as maximum and the control with only SybrSafe as minimum. Low normalized percentage indicates a greater ability to displace SybrSafe from the gDNA and suggests strong binding to gDNA. Three independent experiments were carried out and the results were presented as average values ± standard error of the mean.

### Native Mass Spectrometry

DNA oligonucleotide sequence 5′-CGCATATATGCG-3′ was purchased in lyophilized form (α DNA, Canada) and the purity was confirmed by NMR. Stock solutions of DNA (100 μM) were prepared with 150 mM ammonium acetate buffer solution (Fisher Scientific, Loughborough, Leicestershire, U.K.) and 2 mM potassium chloride solution (Fisher Scientific, Loughborough, Leicestershire, U.K.). This solution was annealed at 90 °C for 10 min and allowed to cool to room temperature. S-MGB stocks (10 mM) in 100% DMSO (Sigma-Aldrich, St. Louis, MO) were diluted to 1 mM S-MGB solution with 150 mM ammonium acetate. Final samples were prepared from this solution to yield final concentrations of 9 μM DNA, 100 μM KCl, and 100 μM S-MGB, 1% DMSO. DNA solutions containing no S-MGB included 1% DMSO and were used as controls.

Native mass spectrometry (nMS) experiments were carried out on a Synapt G2-Si instrument (Waters, Manchester, U.K.) with a nano-electrospray ionization source (nESI). Mass calibration was performed by a separate infusion of NaI cluster ions. Solutions were ionized from a thin-walled borosilicate glass capillary (i.d. 0.78 mm, o.d. 1.0 mm; Sutter Instrument Co., Novato, CA) pulled in-house to nESI tip with a Flaming/Brown micropipette puller (Sutter Instrument Co., Novato, CA). A negative potential in the range of 1.0–1.2 kV was applied to the solution *via* a thin platinum wire (diameter 0.125 mm, Goodfellow, Huntingdon, U.K.). The following instrument parameters were used for the DNA:MGB-BP-3 complex: capillary voltage 1.2 kV, sample cone voltage 80 V, source offset 110 V, source temperature 40 °C, trap collision energy 3.0 V, trap gas 4 mL/min. Data were processed using Masslynx V4.2 and OriginPro 2021, and figures were produced using Chemdraw.

### NMR

DNA samples for NMR spectroscopy were prepared as 1 mM duplex solutions solubilized in 550 μL of a 50 mM phosphate buffer solution prepared with 9:1 H_2_O/D_2_O at pH = 7.4. A concentrated stock solution of MGB-BP-3 was prepared to allow the addition of 5 μL aliquots of solution up to a maximum total addition volume of 50 μL or until a titration end point was detected by virtue of no further changes to the NMR data.

1D ^1^H NMR data were acquired with excitation sculpting for solvent suppression (Bruker pulse program zgesgp). The 600 MHz NMR data were acquired using a Bruker AVANCE-II^+^ NMR spectrometer operating at 600.13 MHz for ^1^H resonance on a standard geometry triple-resonance (TBI-z) probehead equipped for z-pulsed field gradients with the probe temperature regulated and calibrated for data acquisition at 298 K. The 800 MHz NMR data were acquired using a Bruker AVANCE NEO NMR spectrometer operating at 799.43 MHz for ^1^H resonance on a standard geometry 5 mm TCI-z cryoprobe probehead. Sample temperatures were varied within the range of 298–274 K and stabilized prior to data accumulations. In both instances, data were acquired with 128 transients over a ^1^H frequency width equivalent to 20.0276 ppm centered at 4.694 ppm into 32 K (600 MHz) or 64 K (800 MHz) data points using a relaxation delay of 2.0 s between transients. For the excitation sculpting routine, sinc soft pulses (bandwidth ∼125 Hz) were used for selective inversion at the solvent frequency together with smoothed square-shaped gradient pulses (1 ms duration) in a ratio of 31:11. 2D [^1^H, ^1^H] NOESY NMR data were acquired at 800 MHz on the MGB-BP-3/DNA complex stabilized at 274 K (Bruker pulse program noesyesgpph) into 4 K data points for each of 1024 t_1_ increments over ω_1_ and ω_2_ frequency widths equivalent to 20.1756 ppm using 64 transients per increment. A mixing time of 250 ms was applied to allow development of the nOe and chemical exchange responses. Equivalent reference data were acquired for the ligand-free DNA sample at 600 MHz by way of creating a reference data set. All raw data were processed within the TopSpin 4.0.5 environment. Fully processed 2D NMR data were imported into the NMRFAM-Sparky environment (version 1.414 powered by Sparky 3.135^25^) for data interpretation and reduction.

### Enzyme Assays

These experiments were contracted out to Inspiralis Ltd. (Norwich, U.K.). In all experiments, the activity of the enzyme was determined prior to the testing of the compound and 1 U defined as the amount of enzyme required to fully supercoil, decatenate, or relax 0.5 μg of the substrate in 30 min. Cleavage activity was separately determined and 1 U defined as the amount of enzyme that gave maximum cleavage without degradation of the substrate.

MGB-BP-3 was tested over a range from 0.01 to 100 μM (supercoiling, decatenation, and relaxation) or 0.05–500 μM (cleavage) and added to the reaction before the addition of the enzyme. Final DMSO concentration in the assays was 1% (v/v) for supercoiling, decatenation, and relaxation, and 5% for cleavage. All enzymes and DNA substrates used were obtained from Inspiralis Ltd. (Norwich, U.K.). For fluoroquinolone-resistant gyrase supercoiling assays, S83L gyrase for *S. aureus* and S84L gyrase for *E. coli* were used.

#### *E. coli* Gyrase Supercoiling

DNA gyrase (1 U) was incubated with 0.5 μg of relaxed pBR322 DNA in a 30 μL reaction at 37 °C for 30 min under the following conditions: 35 mM Tris–HCl (pH 7.5), 24 mM KCl, 4 mM MgCl_2_, 2 mM DTT, 1.8 mM spermidine, 1 mM ATP, 6.5% (w/v) glycerol, and 0.1 mg/mL BSA. Each reaction was stopped by the addition of 30 μL of chloroform/isoamyl alcohol (24:1) and 20 μL of Stop Dye (40% sucrose, 100 mM Tris·HCl (pH 7.5), 10 mM EDTA, 0.5 μg/mL bromophenol blue) before being loaded on a 1.0% TAE (Tris·acetate 0.04 mM, EDTA 0.002 mM). The gels were run at 90 V for 2 h.

#### *E. coli* Gyrase Cleavage

DNA gyrase (1 U) was incubated with 0.5 μg of supercoiled pBR322 DNA in a 30 μL reaction at 37 °C for 30 min under the following conditions: 35 mM Tris·HCl (pH 7.5), 24 mM KCl, 4 mM MgCl_2_, 2 mM DTT, 1.8 mM spermidine, 6.5% (w/v) glycerol, and 0.1 mg/mL BSA. The reaction was then incubated for a further 30 min with 2% SDS and 0.5 μg/mL proteinase K. Each reaction was stopped by the addition of 30 μL of chloroform/isoamyl alcohol (24:1) and 20 μL of Stop Dye (40% sucrose, 100 mM Tris·HCl (pH 7.5), 10 mM EDTA, 0.5 μg/mL bromophenol blue) before being loaded on a 1.0% TAE (Tris·acetate 0.04 mM, EDTA 0.002 mM) gel, run at 90 V for 2 h.

#### *E. coli* Topo IV Decatenation Assay

Topo IV (1 U) was incubated with 200 ng kDNA (Kinetoplast catenated DNA purified from *Crithidia fasciculata*) in a 30 μL reaction at 37 °C for 30 min under the following conditions: 50 mM HEPES-KOH (pH 7.6), 100 mM potassium glutamate, 10 mM magnesium acetate, 10 mM dithiothreitol, 1 mM ATP, and 50 μg/mL BSA. Each reaction was stopped by the addition of 30 μL of chloroform/isoamyl alcohol (24:1) and 30 μL of Stop Dye before being loaded on a 1.0% TAE gel, run at 90 V for 2 h.

#### *E. coli* Topo IV Relaxation Assay

As for *E. coli* decatenation assay except that the substrate was supercoiled pBR322 DNA.

#### *E. coli* Topo IV Cleavage Assay

Topo IV (1 U) was incubated with 0.5 μg of supercoiled pBR322 DNA in a 30 μL reaction at 37 °C for 30 min under the following conditions: 40 mM HEPES-KOH (pH 7.6), 100 mM potassium glutamate, 10 mM magnesium acetate, 10 mM dithiothreitol, and 50 ug/mL BSA. The reaction was then incubated for a further 30 min with 2% SDS and 0.5 μg/mL proteinase K. Each reaction was stopped by the addition of 30 μL of chloroform/isoamyl alcohol (24:1) and 30 μL of Stop Dye before being loaded on a 1.0% TAE gel, run at 90 V for 2 h.

#### *S. aureus* Gyrase Supercoiling

As for *E. coli* gyrase supercoiling except that the assay conditions were 40 mM HEPES-KOH (pH 7.6), 10 mM magnesium acetate, 10 mM DTT, 2 mM ATP, 500 mM potassium glutamate, and 0.05 mg/mL BSA. The gels were run at 80 V for 3 h.

#### *S. aureus* Gyrase Cleavage

As for *E. coli* gyrase cleavage except that the assay conditions were 40 mM HEPES-KOH (pH 7.6), 10 mM magnesium acetate, 10 mM DTT, 500 mM potassium glutamate, and 0.05 mg/mL BSA. The gels were run at 80 V for 3 h.

#### *S. aureus* Topo IV Decatenation Assay

Topo IV (1 U) was incubated with 200 ng of kDNA in a 30 μL reaction at 37 °C for 30 min under the following conditions: 50 mM Tris·HCl (7.5), 5 mM MgCl_2_, 5 mM DTT, 1.5 mM ATP, 350 mM potassium glutamate, and 0.05 mg/mL BSA. Each reaction was stopped by the addition of 30 μL of chloroform/isoamyl alcohol (24:1) and 30 μL of Stop Dye before being loaded on a 1.0% TAE gel, run at 70 V for 2 h.

#### *S. aureus* Topo IV Cleavage Assay

As for *E. coli* topo IV cleavage except that the assay conditions were 50 mM Tris·HCl (7.5), 5 mM MgCl_2_, 5 mM DTT, 350 mM potassium glutamate, and 0.05 mg/mL BSA.

#### Data Acquisition and Analysis

Bands were visualized by ethidium staining for 10 min, destained for 10 min in water, analyzed by gel documentation equipment (Syngene, Cambridge, U.K.) and quantitated using Syngene Gene Tools software. Raw gel data (fluorescent band volumes) were collected from Syngene, Gene Tools gel analysis software was calculated as a % of the 100% control (fully supercoiled DNA band) and converted to % inhibition. The raw gel data were analyzed using SigmaPlot Version 13 (2015). The global curve fit nonlinear regression tool was used to calculate IC_50_ data using the following equation: Exponential Decay, Single, 2 Parameter *f* = a* exp(−b*x)

### Minimum Inhibitory Concentrations

The minimum inhibitory concentration (MIC) was defined using a microbroth dilution method. Briefly, bacteria at a concentration of 1 × 10^5^ CFU/mL were incubated with doubling dilutions of MGB-BP-3, prepared from a 10 mM DMSO stock, in a 96-well plate for 20 h at 37 °C in tryptic soy broth (Tables 2, 3 and S1) or MHB (Table 1 and Figure 2 and Table S2). Optical density was read and the MIC defined as the lowest concentration of compound to inhibit 80% of visible growth.

For MICs in the presence of membrane permeabilizer and efflux pump inhibitors, PMBN was added at a final concentration of 30 μg/mL (Table 2), and PAβN was added at a final concentration of 100 μg/mL (Table 1) or 25 μg/mL in 0.04 mM MgSO_4_ (Table 2).

Targeted insertion mutants from the *E. coli* Keio knockout collection were maintained on 25 μg/mL kanamycin and MICs were carried out in the presence of 25 μg/mL kanamycin to maintain the kanamycin cassette.

Clinical isolates used in Figure 2 were obtained from NHS Lanarkshire and had antibiotic susceptibility profiles as shown in Table 7.

**Table 7:** Antibiotic Susceptibility Profiles of NHS Clinical Isolates Determined as per 2017 EUCAST Breakpoints^a^

clinical isolate	amoxicillin	co-amoxiclav	tazobactam	ciprofloxacin	gentamicin	doxycycline	cefotaxime	ceftazidime	meropenem	vancomycin
*K. pneumoniae* 1	R	S	S	S	S	NT	S	S	S	R
*K. pneumoniae* 2	R	R	R	R	R	NT	R	R	S	R
*K. pneumoniae3*	R	R	R	R	R	NT	R	R	S	R
*K. pneumoniae* 4	R	S	S	S	S	NT	S	S	S	R
*E. coli* 1	S	S	S	S	S	NT	S	S	S	R
*E. coli* 2	R	R	R	R	R	R	R	R	S	R
*E. coli* 2	R	R	R	R	R	R	R	R	S	R
*E. coli* 4	R	R	S	S	S	NT	S	S	S	R

a R is resistant; S is susceptible; NT is not tested.

### Checkerboard Assays

Dilution series of both MGB-BP-3 and PAβN were prepared in MHB. To evaluate synergy, 25 μL of the MGB-BP-3 solutions were added to wells containing 25 μL of the PAβN solution. To the resulting 50 μL volume of MGB-BP-3 and PAβN was next added 50 μL of 2× bacterial stock, i.e., 2 × 10^5^ CFU/mL (see section Minimum Inhibitory Concentration). After incubation for 20 h at 37 °C, the plates were then transferred to a Tecan Spark plate reader and following a brief shaking (20 s), the optical density of the bacterial suspensions was measured at 600 nm (OD600). The resulting OD600 values were transformed into a 2D gradient to visualize the growth/no-growth results. The FICI was calculated using eq 1, with an FICI ≤ 0.5 indicating synergy^26^
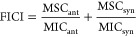
1

eq 1 shows the calculation of FICI where MSC_ant_ = MIC of the antibiotic in combination with synergist; MIC_ant_ = MIC of the antibiotic alone; MSC_syn_ = MIC of the synergist in combination with the antibiotic; and MIC_syn_ = MIC of the synergist alone. In the cases where the MICs were found to exceed the highest concentration tested, the next highest concentration in the dilution series was used in determining the FICI and the result reported as ≤ the calculated value.

### LPS Antagonism Assay

The reaction was carried out as per the Minimum Inhibitory Concentration procedure except that the inoculum was supplemented with 1 μg/mL lipopolysaccharide (LPS) (*E. coli*, Sigma-Aldrich) and a stock solution of 10 mM vancomycin was prepared in sterile water.

### *E. coli* Reporter Strain Assay

The *E. coli* reporter strain assay was carried out as described previously.^18^ Strains from the *E. coli* K12 MG1655 promoter library, maintained on 25 mg/L kanamycin, were treated in supplemented minimal media with subinhibitory concentrations (0.5 × MIC) of compounds at 25 μg/mL kanamycin for 20 h at 37 °C with shaking. MICs for each compound were as follows: MGB-BP-3, 3.125 μM; ciprofloxacin, 2 μg/mL; mitomycin C, 64 mg/L; doxycycline, 4 mg/L. GFP fluorescence and optical density were measured and the fluorescence was normalized to cell density. The fluorescence of the treated samples was divided by the fluorescence of the untreated samples to calculate the fold-induction of the promoter.

### Fluorescence Microscopy

#### Sample Preparation

A 10 mM stock of S-MGB-245 in DMSO, 10 mM stock of PAβN in DMSO, and 100 mM stock of sodium azide in MHB were also prepared. Samples for microscopy were prepared by obtaining log-phase bacteria at a concentration of 1 × 10^5^ CFU/mL in MHB and incubating with 1 μM S-MGB-245, or this and 50 μM PAβN for 1 h. For those experiments with sodium azide, 1 or 10 mM of this was incubated for 1 h before incubation with S-MGB-245. Subsequently, a 10 μL aliquot was mounted on a glass slide and fitted with a cover slip.

#### Image Acquisition

Images were captured using a Zeiss LSM 880 confocal laser scanning microscope and a 63× objective before processing with associated Zen Blue and Adobe Photoshop software. Detection and acquisition parameters were maintained across each species sample set.

## Supporting Information Available

Additional antimicrobial susceptibility data for MGB-BP-3 and fluorescent probe S-MGB-245; details of Boltzmann models from thermal melt experiments; additional mass spectrometry; and NMR data (PDF)

## Notes

I.S.H., C.J.S., and F.J.S. are part of revenue sharing agreements with their University relating to the Strathclyde Minor Groove Binder Project of which MGB-BP-3 is a part. Additionally, I.S.H., C.J.S., and F.J.S. have financial interests through shares in the company, Rostra Therapeutics.

## References

[ref1] Suckling C. J.; Hunter I. S.; Scott F. J. Multitargeted anti-infective drugs: resilience to resistance in the antimicrobial resistance era. Future Drug Discovery 2022, 4, FDD73 10.4155/fdd-2022-0001. 35600289 PMC9112235

[ref2] Kopka M. L.; Yoon C.; Goodsell D.; Pjura P.; Dickerson R. E. The molecular origin of DNA-drug specificity in netropsin and distamycin. Proc. Natl. Acad. Sci. U.S.A. 1985, 82, 1376–1380. 10.1073/pnas.82.5.1376. 2983343 PMC397264

[ref3] Baron R. M.; Lopez-Guzman S.; Riascos D. F.; Macias A. A.; Layne M. D.; Cheng G.; et al. Distamycin A inhibits HMGA1-binding to the P-selectin promoter and attenuates lung and liver inflammation during murine endotoxemia. PLoS One 2010, 5, e10656 10.1371/journal.pone.0010656. 20498830 PMC2871042

[ref4] Brooke D. P.; McGee L. M. C.; Giordani F.; Cross J. M.; Khalaf A. I.; Irving C.; et al. Truncated S-MGBs: towards a parasite-specific and low aggregation chemotype. RSC Med. Chem. 2021, 12, 1391–1401. 10.1039/D1MD00110H. 34447938 PMC8372214

[ref5] Giordani F.; Khalaf A. I.; Gillingwater K.; Munday J. C.; De Koning H. P.; Suckling C. J.; et al. Novel Minor Groove Binders Cure Animal African Trypanosomiasis in an in Vivo Mouse Model. J. Med. Chem. 2019, 62, 3021–3035. 10.1021/acs.jmedchem.8b01847. 30763102

[ref6] Hlaka L.; Rosslee M.-J.; Ozturk M.; Kumar S.; Parihar S. P.; Brombacher F.; et al. Evaluation of minor groove binders (MGBs) as novel anti-mycobacterial agents and the effect of using non-ionic surfactant vesicles as a delivery system to improve their efficacy. J. Antimicrob. Chemother. 2017, 72, 3334–3341. 10.1093/jac/dkx326. 28961913 PMC5890746

[ref7] Kieswetter N. S.; Ozturk M.; Hlaka L.; Chia J. E.; Nichol R. J. O.; Cross J. M.; et al. Intranasally administered S-MGB-364 displays antitubercular activity and modulates the host immune response to *Mycobacterium tuberculosis* infection. J. Antimicrob. Chemother. 2022, 77, 1061–1071. 10.1093/jac/dkac001. 35084027 PMC8969509

[ref8] Nichol R. J. O.; Khalaf A. I.; Sooda K.; Hussain O.; Griffiths H. B. S.; Phillips R.; et al. Selective in vitro anti-cancer activity of non-alkylating minor groove binders. MedChemComm 2019, 10, 1620–1634. 10.1039/C9MD00268E. 32952999 PMC7478159

[ref9] Scott F.; Suckling C. The potential for new and resilient anti-cancer drugs based upon minor groove binders for DNA. Med. Res. Arch. 2021, 9, 2592 10.18103/mra.v9i11.2592.

[ref10] Scott F. J.; Khalaf A. I.; Duffy S.; Avery V. M.; Suckling C. J. Selective anti-malarial minor groove binders. Bioorg. Med. Chem. Lett. 2016, 26, 3326–3329. 10.1016/j.bmcl.2016.05.039. 27212070

[ref11] Scott F. J.; Khalaf A. I.; Giordani F.; Wong P. E.; Duffy S.; Barrett M.; et al. An evaluation of Minor Groove Binders as anti-Trypanosoma brucei brucei therapeutics. Eur. J. Med. Chem. 2016, 116, 116–125. 10.1016/j.ejmech.2016.03.064. 27060763 PMC4872591

[ref12] Scott F. J.; Nichol R. J. O.; Khalaf A. I.; Giordani F.; Gillingwater K.; Ramu S.; et al. An evaluation of Minor Groove Binders as anti-fungal and anti-mycobacterial therapeutics. E. J. Med. Chem. 2017, 136, 561–572. 10.1016/j.ejmech.2017.05.039. 28544982

[ref13] Kerr L.; Browning D. F.; Lemonidis K.; Salih T.; Hunter I. S.; Suckling C. J. Novel antibiotic mode of action by repression of promoter isomerisation. BioRxiV 2020. 10.1101/2020.12.31.424950.

[ref14] Anthony N. G.; Breen D.; Donoghue G.; Khalaf A. I.; Mackay S. P.; Parkinson J. A.; et al. A new synthesis of alkene-containing minor-groove binders and essential hydrogen bonding in binding to DNA and in antibacterial activity. Org. Biomol. Chem. 2009, 7, 1843 10.1039/b901898k. 19590779

[ref15] Lamers R. P.; Cavallari J. F.; Burrows L. L. The Efflux Inhibitor Phenylalanine-Arginine Beta-Naphthylamide (PAβN) Permeabilizes the Outer Membrane of Gram-Negative Bacteria. PLoS ONE 2013, 8, e60666 10.1371/journal.pone.0060666. 23544160 PMC3609863

[ref16] Lomovskaya O.; Warren M. S.; Lee A.; Galazzo J.; Fronko R.; Lee M.; et al. Identification and characterization of inhibitors of multidrug resistance efflux pumps in *Pseudomonas aeruginosa*: novel agents for combination therapy. Antimicrob. Agents Chemother. 2001, 45, 105–116. 10.1128/AAC.45.1.105-116.2001. 11120952 PMC90247

[ref17] Pacios O.; Fernández-García L.; Bleriot I.; Blasco L.; Ambroa A.; López M.; et al. Adaptation of clinical isolates of *Klebsiella pneumoniae* to the combination of niclosamide with the efflux pump inhibitor phenyl-arginine-β-naphthylamide (PaβN): co-resistance to antimicrobials. J. Antimicrob. Chemother. 2022, 77, 1272–1281. 10.1093/jac/dkac044. 35238930

[ref18] Picconi P.; Hind C. K.; Nahar K. S.; Jamshidi S.; Di Maggio L.; Saeed N.; et al. New Broad-Spectrum Antibiotics Containing a Pyrrolobenzodiazepine Ring with Activity against Multidrug-Resistant Gram-Negative Bacteria. J. Med. Chem. 2020, 63, 6941–6958. 10.1021/acs.jmedchem.0c00328. 32515951

[ref19] Baba T.; Ara T.; Hasegawa M.; Takai Y.; Okumura Y.; Baba M.; et al. Construction of *Escherichia coli* K-12 in-frame, single-gene knockout mutants: the Keio collection. Mol. Syst. Biol. 2006, 2, 2006.0008 10.1038/msb4100050. PMC1681482 16738554

[ref20] Parkinson J. A.; Khalaf A. I.; Anthony N. G.; MacKay S. P.; Suckling C. J.; Waigh R. D. Comparison of DNA Complex Formation Behaviour for Two Closely Related Lexitropsin Analogues. Helv. Chim. Acta 2009, 92, 795–822. 10.1002/hlca.200800390.

[ref21] Maxwell A.; Howells A. J. Overexpression and Purification of Bacterial DNA Gyrase. In DNA Topoisomerase Protocols; Springer Nature, 1999; pp 135–144. 10.1385/1-59259-259-7:135 12844869

[ref22] Deweese J. E.; Osheroff N. The DNA cleavage reaction of topoisomerase II: wolf in sheep’s clothing. Nucleic Acids Res. 2009, 37, 738–748. 10.1093/nar/gkn937. 19042970 PMC2647315

[ref23] Holmes N. E.; Charles P. G. P. Safety and Efficacy Review of Doxycycline. Clin. Med. Ther. 2009, 1, CMT.S2035 10.4137/cmt.s2035.

[ref24] Butler M. S.; Gigante V.; Sati H.; Paulin S.; Al-Sulaiman L.; Rex J. H.; et al. Analysis of the Clinical Pipeline of Treatments for Drug-Resistant Bacterial Infections: Despite Progress, More Action Is Needed. Antimicrob. Agents Chemother. 2022, 66, e0199121 10.1128/aac.01991-21. 35007139 PMC8923189

[ref25] Lee W.; Tonelli M.; Markley J. L. NMRFAM-SPARKY: enhanced software for biomolecular NMR spectroscopy. Bioinformatics 2015, 31, 1325–1327. 10.1093/bioinformatics/btu830. 25505092 PMC4393527

[ref26] Odds F. C. Synergy, antagonism, and what the chequerboard puts between them. J. Antimicrob. Chemother. 2003, 52, 1 10.1093/jac/dkg301. 12805255

