# Supplementary material for: Insights into the Spectrum of Activity and Mechanism of Action of MGB-BP-3
Source: ACS Infect Dis. 2022 Nov 29;8(12):2552–63. doi: 10.1021/acsinfecdis.2c00445 (PMC9745797; doi:10.1021/acsinfecdis.2c00445)
Supplement: Supplementary file 1 — id2c00445_si_001.pdf [file id2c00445_si_001.pdf]

Insights into the spectrum of activity and mechanism of action of MGB-BP-3

Electronic Supplementary Information

Charlotte Hind[1], Melanie Clifford[1], Charlotte Woolley[1], Jane Harmer[2], Leah M. C. McGee[3], Izaak Tyson-Hirst[3], Henry J. Tait[3], Daniel P. Brooke[3], Stephanie J. Dancer[4,5], Iain S. Hunter[6], Colin J. Suckling[3], Rebecca Beveridge[3], John A. Parkinson[3], J. Mark Sutton[1,7], Fraser J. Scott[3*]

[1]Research and Evaluation, UKHSA Porton Down, Salisbury, SP4 0JG, United Kingdom
[2]School of Applied Sciences, University of Huddersfield, Queensgate, Huddersfield HD1 3DH, United Kingdom
[3]Department of Pure and Applied Chemistry, University of Strathclyde, Glasgow G1 1XL, United Kingdom
[4]Department of Microbiology, Hairmyres Hospital, NHS Lanarkshire, Glasgow G75 8RG United Kingdom
[5]School of Applied Sciences, Edinburgh Napier University, Edinburgh EH11 4BN, United Kingdom
[6]Strathclyde Institute of Pharmacy & Biomedical Sciences, University of Strathclyde, Glasgow, G4 0RE, United Kingdom
[7]Institute of Pharmaceutical Science, School of Cancer & Pharmaceutical Science, King's College London, Franklin-Wilkins Building, 150 Stamford Street, London SE1 9NH, United Kingdom

***Corresponding Author: fraser.j.scott@strath.ac.uk**

Table of Contents

**Table S1**. MICs of MGB-BP-3 against and expanded panel of Gram-positive.	**Page S1**
**Table S2.** Activity of S-MGB-245 against ESKAPE pathogens.	**Page S1**
**Figure S1:** Assessment of active transport mediated uptake using sodium azide in *S. aureus.*	**Page S1**
**Table S3.** gDNA and gDNA:MGB-BP-3 complex Boltzman model details that relate to figure 4 in main text.	**Page S1**
**Table S4.** Calculated and measured masses for each species observed in Figure 5 for nESI-MS of DNA sequence 5'-CGCATATATGCG-3' MGB-BP-3.	**Page S2**
**Figure S2.** Aromatic cross-peak region of the 250 ms, 800 MHz 2D [^{1}H, ^{1}H] NOESY NMR spectrum acquired at 1 °C of duplex d(CGCATATATGCG)$_2$ in complex with 2 molar equivalents of MGB-BP-3.	**Page S2**
**Table S5.** 1H NMR chemical shift assignments for identified protons within the DNA duplex of sequence 5'-d(CGCATATATGCG)-3' in ligand-bound form	**Page S3**
**Table S6.** Comparisons of 1H NMR chemical shift data for ligand-free compared with ligand-bound DNA duplex of sequence 5'-(CGCATATATGCG)-3' for assignable resonances within the ligand-bound complex for identifiably distinct DNA strands with the ligand-bound form.	**Page S4**

Table S1. MICs of MGB-BP-3 against and expanded panel of Gram-positive. Ciprofloxacin (CIP) was included as a control antibiotic.

Organism	Strain	MGB-BP-3	CIP
S. aureus	ATCC 9144	0.0977	1
S. aureus	NCTC 13616	0.39	64
S. aureus	USA300	0.195	64
S. aureus	1199b	0.39	8
E. faecalis	NCTC 775	0.39	2
E. faecalis	NCTC 12201	0.78	1
E. faecium	NCTC 12204	0.39	1

Table S2. Activity of S-MGB-245 against ESKAPE pathogens and potentiation results with PAβN against Gram-negative pathogens.

	S. aureus ATCC 43300	*E. faecalis* ATCC 51299	*E.coli* ATCC 25922	*P. aeruginosa* ATCC 27893	*A. baumannii* ATCC 19606	*K. pneumoniae* ATCC 700603
**MIC_{80} (µM) S-MGB-245**	1.56	6.25	>100	>100	>100	>100
**MIC_{80} (µM) S-MGB-245 with 100 µg/mL PAβN**	NT	NT	3.13	6.25	3.13	12.5

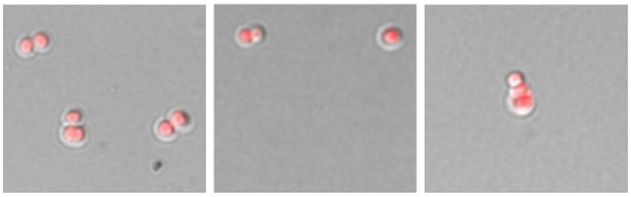

Figure S1: Assessment of active transport mediated uptake using sodium azide in *S. aureus*

Table S3. gDNA and gDNA:MGB-BP-3 complex Boltzman model details that relate to figure 4 in main text.

	Model	Equation	A1	A2	X_0	dx	Reduced Chi-Sqr	R-Square (COD)	Adj. R-Square
**gDNA: MGB-BP-3 Complex**	Boltzmann	y = A2 + (A1-A2)/(1 + exp((x-x0)/dx))	0.01947 ± 0.00405	0.97237 ± 0.00661	81.46442 ± 0.06105	1.20681 ± 0.05292	7.28691E-4	0.99597	0.99581
**gDNA**	Boltzmann	y = A2 + (A1-A2)/(1 + exp((x-x0)/dx))	-0.02267 ± 0.01116	0.92405 ± 0.00639	69.97987 ± 0.1574	3.44394 ± 0.14232	8.89592E-4	0.9938	0.99355

Table S4. Calculated and measured masses for each species observed in Figure 5 for nESI-MS of DNA sequence 5'-CGCATATATGCG-3' MGB-BP-3.

Species	m/z value	Calculated mass of neutral species (Da)
Single Stranded [SS]	3- : 1214.1<br>4- : 910.3	(1214.1*3) + 3 = 3645.3<br>(910.3*4) + 4 = 3645.2
Double Stranded [DS]	4- : 1821.7<br>5- : 1457.2	(1821.7*4) + 4 = 7290.8<br>(1457.2*5) + 5 = 7291.0
Double Stranded + 2 x S-MGB-BP-3 [DS+2M]	4- : 2137.2<br>5- : 1709.6	(2137.2) + 4 = 8552.8<br>(1709.6*5) +5 = 8553.0

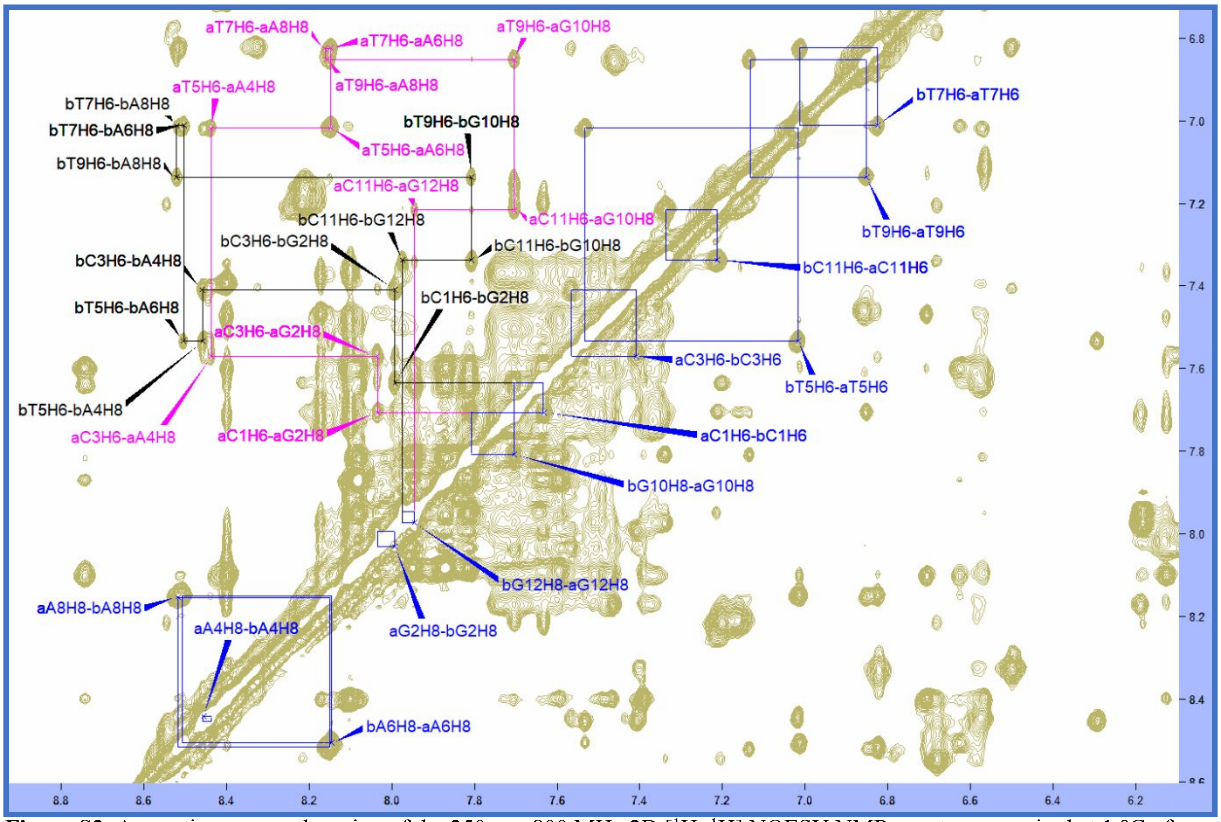

Figure S2. Aromatic cross-peak region of the 250 ms, 800 MHz 2D [^{1}H, ^{1}H] NOESY NMR spectrum acquired at 1 °C of duplex d(CGCATATATGCG)$_2$ in complex with 2 molar equivalents of MGB-BP-3 showing the 5'-3' assignment "walk" and evidence of two interchanging identities for the DNA strands. Magenta lines and labels – DNA strand "a" assignments showing nOe correlations between aromatic protons within nucleotides adjacent to one another; Black lines and labels – as for DNA strand "a" but for DNA strand "b" instead; Blue squares and labels – cross-peaks arising from a chemical exchange process which swaps the identities of proton nuclei in Strand "a" and Strand "b". Assignments are read as: nXcHd where n is "a" or "b" for DNA strands, X is the nucleotide type A, G, C or T, c is the nucleotide number within the sequence counting from the 5' terminal base as 1; Hd is the identity of the relevant aromatic proton.

Table S5. ^1H NMR chemical shift assignments for identified protons within the DNA duplex of sequence 5'-d(CGCATATATGCG)-3' in ligand-bound form.

	Chemical Shift Assignment: δ ^1H (ppm)[a]														
**Atom ID**	H8			H6			H5			CH_3			H1'		
	**a**[b]	**b**[b]	Δδ[c]	**a**	**b**	Δδ	**a**	**b**	Δδ	**a**	**b**	Δδ	**a**	**b**	Δδ
**Base**															
C1				7.704	7.634	0.070	5.935	5.895	0.04				5.778	5.740	0.038
G2	8.035	7.992	0.043										6.105	5.867	**0.238**
C3				7.568	7.409	**0.159**	5.597	5.438	**0.159**				5.747	5.826	-0.079
A4	8.438	8.458	-0.020										6.574	6.292	**0.282**
T5				7.018	7.533	**-0.515**				1.570	1.520	0.050	5.378	5.509	**-0.131**
A6	8.148	8.505	**-0.357**										6.626	6.625	0.001
T7				6.824	7.012	**-0.188**				1.342	1.647	**-0.305**	5.323	5.369	-0.046
A8	8.155	8.519	**-0.364**										5.184	5.490	**-0.306**
T9				6.852	7.135	**-0.283**				1.436	1.476	-0.040	5.328	5.249	0.079
G10	7.704	7.808	**-0.104**										5.931	5.925	0.006
C11				7.215	7.338	**-0.123**	5.258	5.369	**-0.111**				5.656	5.648	0.008
G12	7.946	7.975	-0.029										6.185	6.185	0.000

[a] ^1H NMR data assignments for data acquired on a sample cooled to 1 °C. [b] Chemical exchange results in two identifiable sets of resonances arising from DNA strands having different, yet interconverting identities here termed **a** and **b**. [c] Δδ corresponds to the difference between ^1H chemical shift values of resonances associated with the "**a**" strand compared with resonances associated with the "**b**" strand of the DNA as $\Delta\delta = \delta^1H_a - \delta^1H_b$ in all cases of calculated proton chemical shift differences. Colour coding: chemical shift difference values are colour coded blue or red where red corresponds to base proton values and blue corresponds to sugar proton values. Bold typeface entries are for the most significant differences where |Δδ| > 0.1 ppm.

Table S6. Comparisons of ^{1}H NMR chemical shift data for ligand-free compared with ligand-bound DNA duplex of sequence 5'-(CGCATATATGCG)-3' for assignable resonances within the ligand-bound complex for identifiably distinct DNA strands with the ligand-bound form.

**Chemical Shift Assignment: $\delta^{1}H$ (ppm)**															
**Atom ID**	H8			H6			H5			CH_3			H1'		
	Free (f)[a]	$\Delta\delta_{(a\text{-}f)}$[b]	$\Delta\delta_{(b\text{-}f)}$[b]	Free (f)	$\Delta\delta_{(a\text{-}f)}$	$\Delta\delta_{(b\text{-}f)}$	Free (f)	$\Delta\delta_{(a\text{-}f)}$	$\Delta\delta_{(b\text{-}f)}$	Free (f)	$\Delta\delta_{(a\text{-}f)}$	$\Delta\delta_{(b\text{-}f)}$	Free (f)	$\Delta\delta_{(a\text{-}f)}$	$\Delta\delta_{(b\text{-}f)}$
**Base**															
C1				7.654	0.005	-0.020	5.911	0.024	-0.016				5.760	0.018	-0.020
G2	7.976	0.059	0.016										5.916	**0.189**	-0.049
C3				7.413	**0.155**	-0.004	5.453	**0.144**	-0.015				5.593	**0.154**	**0.233**
A4	8.363	0.075	0.095										6.288	**0.286**	0.004
T5				7.204	**-0.186**	**0.328**				1.481	0.089	0.039	5.685	**-0.307**	**-0.176**
A6	8.275	**-0.127**	**0.230**										6.240	**0.386**	**0.385**
T7				7.206	**-0.382**	**-0.194**				1.354	-0.012	**0.293**	5.802	**-0.479**	**-0.433**
A8	8.272	**-0.117**	**0.247**										6.238	**-1.054**	**-0.748**
T9				7.117	**-0.265**	0.018				1.298	**0.138**	**0.178**	5.822	**-0.494**	**-0.573**
G10	7.883	**-0.179**	-0.075										5.803	**0.128**	**0.122**
C11				7.380	**-0.166**	-0.042	5.420	**-0.162**	-0.051				5.761	**-0.105**	**-0.133**
G12	7.980	-0.034	-0.005										6.181	0.004	0.004

[a] ^{1}H NMR data assignments for protons in the ligand free DNA duplex. [b] Chemical exchange results in two identifiable sets of resonances arising from DNA strands having different, yet interconverting identities here termed **a** and **b**. $\Delta\delta_{(a\text{-}f)}$ corresponds to the difference between ^{1}H chemical shift values of resonances associated with ligand-free DNA duplex subtracted from the ligand-bound "**a**" strand as $\Delta\delta = \delta^{1}H_{a} - \delta^{1}H_{f}$. $\Delta\delta_{(b\text{-}f)}$ corresponds to the difference between ^{1}H chemical shift values of resonances associated with ligand-free DNA duplex subtracted from the ligand-bound "**b**" strand as $\Delta\delta = \delta^{1}H_{b} - \delta^{1}H_{f}$. Colour coding: chemical shift difference values are colour coded blue or red where red corresponds to base proton values and blue corresponds to sugar proton values. Bold typeface entries are for the most significant differences where $|\Delta\delta| > 0.1$ ppm.
